# Epoxy- versus Glutaraldehyde-Treated Bovine Jugular Vein Conduit for Pulmonary Valve Replacement: A Comparison of Morphological Changes in a Pig Model

**DOI:** 10.3390/biomedicines11113101

**Published:** 2023-11-20

**Authors:** Nataliya R. Nichay, Anna A. Dokuchaeva, Yuriy Yu. Kulyabin, Evgeniy V. Boyarkin, Elena V. Kuznetsova, Yanina L. Rusakova, Ivan S. Murashov, Andrey A. Vaver, Alexander V. Bogachev-Prokophiev, Irina Yu. Zhuravleva

**Affiliations:** 1 E. Meshalkin National Medical Research Center, Ministry of Health of Russian Federation, 15 Rechkunovskaya St., Novosibirsk 630055, Russia; a_dokuchaeva@meshalkin.ru (A.A.D.); ju_kuljabin@meshalkin.ru (Y.Y.K.); e_bojarkin@meshalkin.ru (E.V.B.); ev_kuznetsova@meshalkin.ru (E.V.K.); y_rusakova@meshalkin.ru (Y.L.R.); i_murashov@meshalkin.ru (I.S.M.); vaver_a@meshalkin.ru (A.A.V.); a_bogachev@meshalkin.ru (A.V.B.-P.); zuravleva_i@meshalkin.ru (I.Y.Z.); 2 Cardiovascular Department, Novosibirsk State Medical University, Ministry of Health of Russian Federation, 52 Krasny Prospect, Novosibirsk 630091, Russia

**Keywords:** bovine jugular vein, Contegra, diepoxide, glutaraldehyde, pulmonary conduit, right ventricular outflow tract reconstruction

## Abstract

Valved conduits are often required to replace pulmonary arteries (PA). A widely used Contegra device is made of bovine jugular vein (BJV), preserved with glutaraldehyde (GA) and iso-propanol. However, it has several drawbacks that may be attributed to its chemical treatment. We hypothesized that the use of an alternative preservation compound may significantly improve BJV conduit performance. This study aimed to compare the macroscopic and microscopic properties of the BJV treated with diepoxide (DE) and GA in a porcine model. Twelve DE-BJVs and four Contegra conduits were used for PA replacement in minipigs. To assess the isolated influence of GA, we included an additional control group—BJV treated with 0.625% GA (n = 4). The animals were withdrawn after 6 months of follow-up and the conduits were examined. Explanted DE-BJV had a soft elastic wall with no signs of thrombosis or calcification and good conduit integration, including myofibroblast germination, an ingrowth of soft connective tissue formations and remarkable neoangiogenesis. The inner surface of DE-BJVs was covered by a thin neointimal layer with a solid endothelium. Contegra grafts had a stiffer wall with thrombosis on the leaflets. Calcified foci, chondroid metaplasia, and hyalinosis were observed within the wall. The distal anastomotic sites had hyperplastic neointima, partially covered with the endothelium. The wall of GA-BJV was stiff and rigid with degenerative changes, a substantial amount of calcium deposits and dense fibrotic formations in adventitia. An irregular neointimal layer was presented in the anastomotic sites without endothelial cover in the GA BJV wall. These results demonstrate that DE treatment improves conduit integration and the endothelialization of the inner surface while preventing the mineralization of the BJV, which may reduce the risk of early conduit dysfunction.

## 1. Introduction

Valved conduits are often applied for right ventricular outflow tract (RVOT) reconstruction, particularly in children with congenital heart disease (CHD). A Contegra^®^ conduit is widely used as the right ventricle–pulmonary artery (RV-PA) conduit. The Contegra pulmonary valved conduit is made of bovine jugular vein (BJV) with a venous valve, preserved and stored in 1% glutaraldehyde (GA) and 20% isopropyl alcohol. The benefit of such a xenograft is a natural three-leaflet valve, while the venous wall conduit is sufficient for low-pressure flow conditions. Another advantage of the Contegra conduit is the availability of various sizes, and especially small sizes. Several studies demonstrated that postoperative performance of Contegra is comparable to that of pulmonary homografts [1,2,3]. However, Contegra is prone to neointimal proliferation with the formation of predominantly distal stenoses as well as progressive regurgitation due to conduit dilatation, calcification, and thrombosis, which may affect its performance [1,3,4,5,6,7]. Also, Contegra may be more susceptible to infective endocarditis in comparison to homografts and some bioprostheses [2,8,9].

One of the main causes of degenerative changes within the wall and leaflets of the Contegra conduit is GA cross-linking. The disadvantages of GA treatment, such as cytotoxicity, rapid calcification, a predisposition to neointimal proliferation, fibrosis and thrombosis are well established [10,11,12,13,14]. Compared to GA, epoxy treatment reduces the degree of calcium accumulation, immunogenicity, and cytotoxicity of the biomaterial, while not significantly affecting the mechanical properties of the tissue [10,15,16,17,18,19].

Our team has substantial experience in RVOT reconstruction using diepoxy-treated (ethylene glycol diglycidyl ether, DE) conduits. DE-treated bovine pericardium grafts show good performance in adult patients and adolescents [20,21], providing greater freedom from conduit dysfunction in midterm follow-up as well as a lower tendency for neointimal proliferation in comparison to that in GA-treated conduits [22]. However, the use of bovine pericardium grafts in the pediatric population is limited due to their large size. BJV conduits seem more suitable for children. We suggest that switching the BJV treatment from GA to DE may improve conduit performance. To the best of our knowledge, DE treatment has not been used for BJV conduits so far. This is the first study evaluating diepoxy-treated bovine jugular vein (DE-BJV) performance in pulmonary circulation.

In this study, we aimed to compare the performance and structural changes of a DE-BJV conduit versus the Contegra graft in a large animal model with a 6-month follow-up. We included an additional control group, BJV treated with 0.625% GA, in order to assess the isolated influence of GA on the macroscopic and histological structures of the BJV.

## 2. Materials and Methods

### 2.1. Study Design

The local ethics committee approved this study. All experimental procedures were performed in compliance with the local and international regulations and standards of animal care.

**Figure 1:**
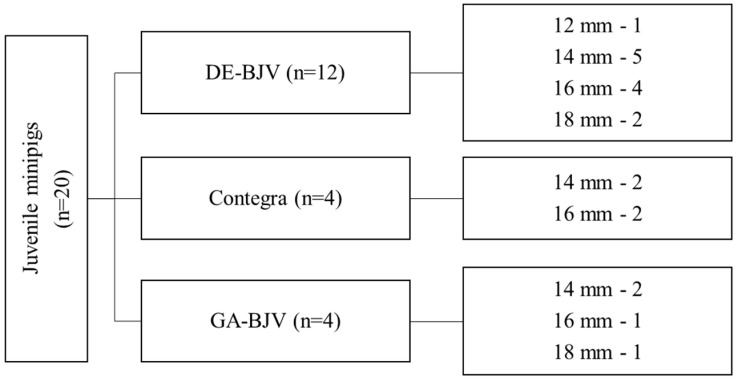
Study design. DE-BJV, diepoxy-treated bovine jugular vein; GA-BJV, glutaraldehyde-treated bovine jugular vein.

This study included 20 healthy animals (ICG minipigs [23], Institute of Cytology and Genetics, Novosibirsk, Russia) weighing 33–72 kg (Table 1). Twelve pigs underwent implantation of the DE-BJV conduits (Figure 1). The eight remaining pigs were equally divided into groups with implanted Contegra conduits (Contegra pulmonary valved conduit; Medtronic, Minneapolis, MN, USA) and with an implanted BJV treated with 0.625% GA (GA-BJV). In all animals, the main pulmonary artery (PA) was grafted with a BJV conduit under cardiopulmonary bypass (CPB). The follow-up period was 6 months. The animals were euthanized from the experiment at the end of the 6-month observation period.

### 2.2. Preparation of the Conduit

As a cross-linking agent, we used 25% GA (catalog No. 253857, Panreac Quimica SLU, Barcelona, Spain) and 97% DE, commercially available from N. Vorozhtsov Novosibirsk Institute of Organic Chemistry, SB RAS (Novosibirsk, Russian Federation).

Fresh BJVs with natural valves were extracted from healthy animals immediately after slaughter and rinsed several times with 0.9% NaCl. Hydraulic tests and endoscopic examinations (Hopkins endoscope; 30°; diameter, 4 mm; Karl Storz SE & Co. KG, Tuttlingen, Germany) were used to assess venous valve competence. 

Selected BJVs with a competent valve were preserved using one of two different ways:

(1) For 14 days at room temperature in 5% buffered (0.05 M phosphate buffer, pH 7.4) DE solution, changed once, on day 2;
(2) For 14 days at room temperature in 0.625% buffered (0.05 M phosphate buffer, pH 7.4) GA solution, with conservative replacement on days 2, 4, and 8.

After preservation, the conduits were stored individually in sealed, sterile packages in a biocidal solution of the original composition [24].

The control group was a Contegra graft (Contegra pulmonary valved conduit; Medtronic, Minneapolis, MN, USA), a product commercially available for clinical use. According to the manufacturer’s instructions, it consists of a xenogeneic (bovine) jugular vein with a trileaflet venous valve and a natural sinus slightly larger in diameter than its lumen. A final sterilization step was performed using a proprietary sterilizer containing 1% GA and 20% isopropyl alcohol, in which the conduit was preserved and packaged until use.

### 2.3. Experimental Conduit Implantation

#### 2.3.1. Anesthesia and Mechanical Ventilation

An anesthesiologist and veterinarian with good laboratory practice qualifications jointly carried out anesthetic management at all stages. Before the operation, the animals were examined by a veterinarian. Twelve hours before surgery, the animals were given access only to water, and were deprived of food. An hour before the start of the intervention, after confirmation by the veterinarian of a satisfactory condition, the animal was premedicated with Zoletil-100 (Virbac Sante Animale, France) 5–7 mg/kg intramuscularly. Upon reaching the target level of sedation and after the scrubbing of surgical sites, the animal was transported to the operating room and fixed on the operating table in the supine position. After preoxygenation, anesthesia was supplemented with fentanyl (6–8 µg/kg), propofol (4–6 mg/kg) and pipecuronium bromide (0.1 mg/kg). The animal was intubated and ventilated at O_2_ 50% vol., at a tidal volume of 6–8 mL/kg, with a positive end-expiratory pressure of 8 cm H_2_O, maintaining PaCO_2_ at 36–43 mmHg (JulianPlus ventilator, Draege, Germany). In the ventilated animal, anesthesia was maintained using sevoflurane 2–4 vol% and intravenous fentanyl 1–2 µg/kg every 20 min. An invasive arterial blood line was placed in the femoral artery, and a central venous catheter was placed into the femoral vein for additional infusions and blood sampling. A urinary catheter was installed to control urine output. During cardiopulmonary bypass, anesthesia was maintained via the constant IV infusion of propofol (6–10 mg/kg/h) and the bolus administration of fentanyl 1–4 µg/kg every 20–30 min. Cefazolin (2.0 g) was used as the antibiotic prophylaxis. In the postperfusion period, in the presence of signs of heart failure, inotropic support with dopamine infusion was utilized. In our study, dopamine infusion (5 μg/kg/min) was required in one animal during CPB weaning and decannulation. In the postoperative period and restoration of spontaneous breathing, in the absence of signs of heart failure and satisfactory oxygenation (saturation > 90%, PaO_2_ > 70 mmHg, PaCO_2_ 33–45 mmHg), the animal was extubated, observed for 30–60 min and transported to the vivarium in an individual aviary.

#### 2.3.2. Cardiopulmonary Bypass

Before the cannulation of the great vessels, the pigs were heparinized (300 IU/kg). During cardiopulmonary bypass, the activated clotting time (ACT) was maintained at 500–600 s. A MAQUET HL 20 heart–lung machine (MAQUET Cardiopulmonary AG, Rastatt, Germany), a HCU 30 thermoregulator (MAQUET Cardiopulmonary AG, Germany), and Quadrox oxygenators (MAQUET Cardiopulmonary AG, Germany) were used during the experiment. The volumetric perfusion rate was calculated as 60–70 mL per kg of the animal’s body weight. Systemic arterial pressure was maintained at 90–110 mmHg. To ensure adequate venous flow, we used a VAVD MAQUET HL 20 vacuum venous outflow controller (MAQUET Cardiopulmonary AG, Germany) with a negative pressure of 30–50 mmHg. During perfusion, body temperature was maintained at 37–37.5 °C. The hematocrit level was in the range of 24–30%. After venous and arterial decannulation, protamine (300 IU/kg) was administered.

#### 2.3.3. Operative Techniques

The surgical approach was performed through a left lateral thoracotomy via the 4th intercostal space. After systemic heparinization, the great vessels were cannulated, and CPB was initiated. Cannulation was performed centrally through thoracotomy (n = 12) or peripheral vessels (n = 8) depending on the surgical preferences and weight of the animal. The size of the conduit was selected according to the weight of the animal and the diameter of the native main PA. 

**Figure 2:**
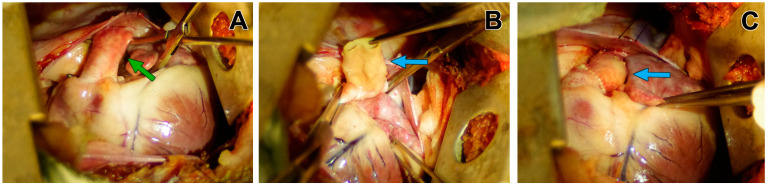
Implantation of a conduit in the pulmonary position: (**A**) left thoracotomy and pulmonary trunk mobilization (the green arrow); (**B**) graft implantation, and the creation of proximal anastomosis between the PA and BJV conduit, where the blue arrow indicates the BJV conduit; (**C**) a BJV conduit in the pulmonary position, where the blue arrow indicates the BJV conduit.

Before implantation, the conduit was washed from the storage solution in a sterile 0.9% sodium chloride solution with three changes of the solution every 20 min for DE-BJV or GA-BJV, and every 5 min for Contegra (in accordance with manufacturer’s recommendations). Conduit implantation was performed on the beating heart and a normothermic full-flow CPB. After the transection of the native PA (Figure 2A), the conduit was implanted orthotopically, just above the native pulmonary valve, after the leaflets were excised. Continuous sutures were used for both the proximal and distal anastomoses (Figure 2B,C). In all cases, double sterility control was performed before the implantation.

#### 2.3.4. Postoperative Management

In the first 7 days of the postoperative period, the animals received nadroparin calcium (0.3 mL 2 times a day), followed by antiplatelet therapy (clopidogrel 75 mg 1 time a day and acetylsalicylic acid 75 mg 1 time a day) during the entire observation period. Amoxicillin clavulanate (8.75 mg/kg; Synulox, Haupt Pharma Latina S.R.L., Borgo San Michele, Latina, Italy) was used for antibiotic prophylaxis. At the end of the 6-month observation period, the animals were withdrawn from the experiment via the application of super-therapeutic doses of sodium thiopental after preliminary sedation (Zoletil-100 5–7 mg/kg).

### 2.4. Macroscopic and Microscopic Study of the Conduits

The BJV conduits were excised from the adjacent native tissues of the right ventricle (RV) and PA. Macroscopic assessments of the conduit wall, cusps, sinuses of the valve, and adjacent fragments of the native PA were performed to determine thrombus formation, calcification, obvious stenosis, or aneurysm formation. Histological specimens were obtained by dissecting the conduit fragments with valves and both anastomoses along the entire length, with subsequent preservation in a 10% buffered formalin solution. Before examination, the tissues were dehydrated and embedded in paraffin; 6-micron sections were stained with hematoxylin and eosin (H&E), von Kossa, Russel-Movat pentachrome stains and an immunohistochemical (IHC) stain for S 100 proteins.

### 2.5. Scanning Electronic Microscopy (SEM)

Briefly, 6 µm sections of each sample were dried at room temperature, straightened, and fixed on the specimen stub. Before the study, the samples were covered with a 25–30 nm thick conductive carbon layer on GVC-3000 Thermal Evaporation Carbon Plating Instrument (KYKY TECHNOLOGY Co., Ltd., Beijing, China). SEM and energy-dispersive X-ray spectroscopy (EDS) analyses of the cell-containing samples and an elemental mapping of the chosen areas were performed using a WIN SEM A6000LV scanning electron microscope (KYKY TECHNOLOGY Co., Ltd., Beijing, China) equipped with an AzTec One EDX system (Oxford Instruments, High Wycombe, UK). Sample observation was conducted using a secondary electron detector at an electron high tension of 20 keV and with an electron beam setting of 120 µA. Ten observation fields were selected for each specimen and examined at a 100×, 250×, 450× and 700× magnification. The sizes of the observed objects were measured using the Microsoft KYKY SEM software (version 1.8.1.2).

### 2.6. Statistical Analyses

Continuous data are reported as median (Me) and interquartile range (IQR), and categorical data are reported as rates and percentages. Descriptive statistics were obtained using STATA version 13.0 (StataCorp LP, College Station, TX, USA).

## 3. Results

All conduits were successfully implanted, without any surgical complications. All animals survived the procedure and were extubated 1.5–3 h postoperatively. Four pigs did not survive for 6 months. One animal from the Contegra group died 1.6 months after the procedure due to an unrelated cause (Dilatation ventriculi acuta). Three animals had prosthetic endocarditis (DE-BJV—1; Contegra—1; GA-BJV—1) and all of them were euthanized after 5.8, 4.8, and 3.2 months, respectively. The other animals survived until the end of the follow-up period.

### 3.1. Macroscopic Findings

**Figure 3:**
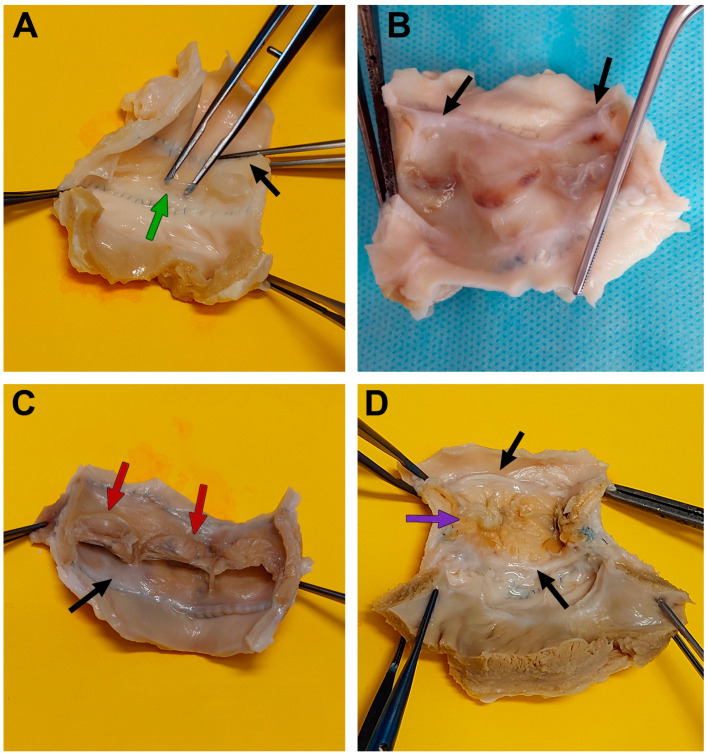
Macrophotographs of explanted conduits after 6 months of follow-up. (**A**) A DE-BJV; the black arrow indicates a thin neointimal layer, and the green arrow indicates intact valve leaflets. (**B**) A DE-BJV; the black arrow indicates neointimal hyperplasia. (**C**) A Contegra graft; the black arrow indicates thick neointima, and red arrows indicate fibrin and thrombotic masses. (**D**) A GA-BJV; the black arrow indicates substantial neointimal hyperplasia, and the purple arrow indicates a deformed leaflet, partially fused with the conduit wall.

The DE-BJV conduits were well incorporated at both the proximal and distal ends, without distortion or aneurysmal formation. All grafts maintained their extensibility, elasticity, and softness. Clean, smooth, white luminal surfaces without thrombus deposition or calcified lesions were observed (Figure 3A). A thin neointimal layer was observed on the inner surface of the conduit walls. The leaflets were intact, soft, and mobile without calcification, fenestration, or tears. In two grafts, intimal hyperplasia was observed along the suture line of the distal anastomosis (Figure 3B). Neointimal proliferation was most pronounced along the lesser curvature of the conduit. One DE-BJV conduit (12 mm) showed a significant size mismatch between the main PA and the RVOT due to the fast growth of the animal during the follow-up period.

Contegra conduits had more rigid walls; however, they showed sufficient pliability. All the conduits had patent lumens (Figure 3C). The wall of the Contegra graft was covered by a fibrotic layer. Two conduits had neointimal hyperplasia at the distal anastomotic site with the greatest thickness along the smaller curve (the first was explanted after 1.6 months, and the second was explanted after 6 months of follow-up). Thick calcified areas were found at both the distal and proximal anastomotic sites. The cusps were thin, mobile, and uncalcified. A thrombotic mass and fibrin deposition were found on the inner surface of the cusps and sinuses of the two conduits.

The wall of the GA-BJV was stiff and rigid, and the inner surface was covered with fibrous tissue. In one case, the wall was loose and wrinkled, with a partially exfoliated neointimal layer. Two conduits had deformed cusps that were partially fused to the conduit wall (Figure 3D). Neointimal hyperplasia and areas of calcified lesions were found at both the proximal and distal anastomotic sites.

In cases of infectious endocarditis, the process was localized strictly at the level of the conduit, without going beyond the suture margins.

### 3.2. Microscopic Examination

**Figure 4:**
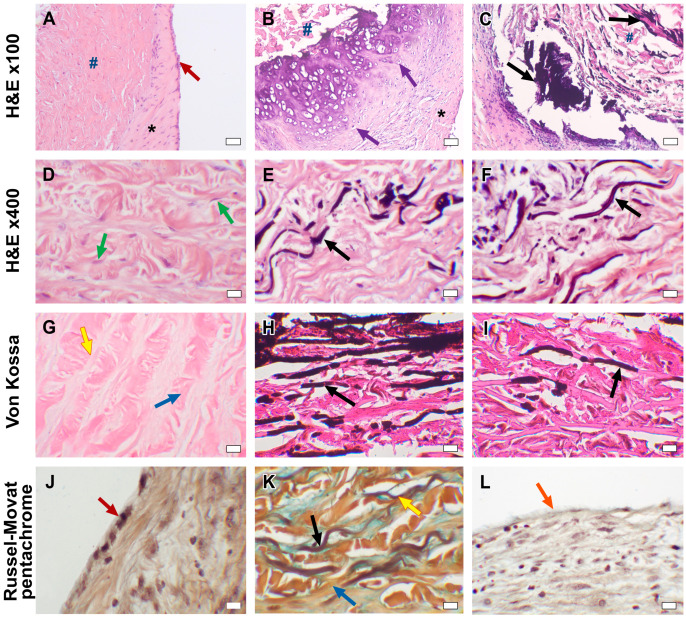
Microphotographs of explanted conduits after 6 months of follow-up: (**A**–**C**) H&E overview images; bars are 50 μm. (**D**–**F**) H&E, conduit wall structure, bars 10 μm. (**G**–**I**) von Kossa staining, ×400; bars are 10 μm. (**J**–**L**) Russel–Movat pentachrome staining ×400; bars are 10 μm, where black arrows indicate mineralization sites, while green arrows indicate migrating fibroblasts, yellow arrows indicate elastin fibers, blue arrows indicate collagen fibers, purple arrows indicate hyalinosis and chondroid metaplasia, red arrows indicate an endothelial monolayer and the orange arrow indicates the absence of an endothelium. *—neointima; #—conduit tissue.

In DE-BJV conduits, the wall preserved its own structure. The elastic and collagen fibers were correctly directed, and small focal fragmentation and disorganization of the collagen fibers were observed in some areas with no specific localization. We discovered the remodeling of the conduit tissue in the form of wall germination by myofibroblast cells from the adventitial side into the medial layer, the replacement of collagen fibers with soft non-deforming connective tissue, and areas of neoangiogenesis (Figure 4A,D). In some conduits, the infiltration of inflammatory cells (lymphocytes; granulocytes) was observed in various areas.

A uniform neointimal layer covering the inner surface of the DE-BJV originated from the side of both the proximal and distal anastomoses and did not involve the cusps. The thickness of the neointimal layer was in the range of ~110–320 µm in the central part of the conduit and ~130–620 µm near the anastomoses. Fibroblasts were equally distributed in the fibrous sheath. The well-developed endothelial monolayer extended from the PA and fully covered the fibrous tissue layer (Figure 4A,J).

**Figure 5:**
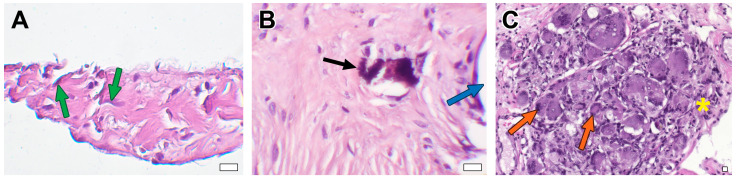
Microphotographs of DE-BJV graft; H&E staining: (**A**) Valve leaflet; green arrows indicate migrating fibroblasts. (**B**) Site of mineralization; black arrows indicate mineral clusters and the blue arrow indicates suture hole; (**C**) A giant cell granuloma; orange arrows indicate giant cells, and the yellow asterisk indicates granuloma tissue. Magnification, ×400; bar, 10 μm.

No signs of tissue mineralization or calcium deposition in the elastin or collagen fibers were found in the DE-BJV conduits (Figure 4G). Small dense calcium clusters were present around some stitches along the suture line, both in the graft tissue and the PA tissue in some conduits (n = 4) (Figure 5B).

The structure of the cusps was preserved in all cases. Collagen and elastin fibers were well-preserved in the leaflets. The migration of myofibroblastic cells into the cusps was observed in some conduits (Figure 5A). Calcification was not observed in the leaflets. No endothelial cells were present on either the inflow or outflow valve surfaces.

In two DE-BJV conduits, dense hyperplastic neointima was found in the area of the distal suture line (thickness range, ~1250–1800 µm). A large number of multinucleated macrophages forming giant-cell granulomas were present in the adventitial layer at the distal anastomotic site of the conduit (Figure 5C). Neovascularization and a dense fibrous tissue with inflammatory cells consisting of lymphocytes and histiocytes were observed around the giant-cell granulomas. At the sites of adventitial fibrosis, the deformation of the conduit wall occurred due to the formation of an adventitial scar of dense fibrous tissue. The accumulation of giant-cell macrophages in the subneointimal region of these grafts was also observed.

**Figure 6:**
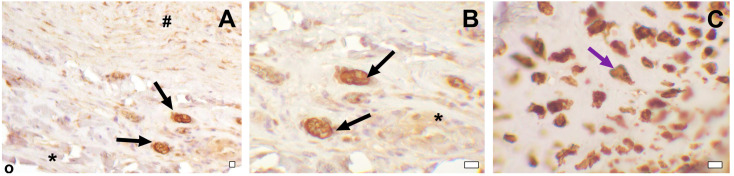
IHC staining for S100 proteins. (**A**) Contegra; luminal surface; magnification, ×100. (**B**) Contegra; luminal surface; magnification, ×400. (**C**) Hyalinosis in anastomosis area. Black arrows indicate subintimally located S100-positive cells, showing signs of chondroid metaplasia, and the purple arrow indicates S100-positive cells inside a focus of hyalinosis. *—neointima; #—conduit tissue; °—graft lumen. Bars—10 μm.

The explanted Contegra grafts maintained their native layer structure; collagen and elastin fibers retained the correct orientation. A focal fragmentation of the collagen fibers was observed. Large calcium deposits and areas of calcification were revealed at the anastomotic sites. A significant accumulation of calcium in the elastin fibers was found throughout the wall of the conduit, with a predominance in the subintimal layer (Figure 4H,K). Calcified elastin fibers were also present in the conduit explanted after 1.6 months of follow-up (Figure 4E). Three out of four Contegra conduits showed hyalinosis with signs of mineralization and subintimal sites of chondroid metaplasia in the graft wall (Figure 4B). Signs of chondroid metaplasia, hyalinosis and mineralization were revealed in the PA tissue at the anastomotic site. Cells demonstrating chondroid metaplasia showed positive IHC staining for S 100 proteins (Figure 6). Inflammatory cells, mainly lymphocytes, neutrophils, and single multinucleated macrophages, were present in the tunica adventitia of the conduits. The inflammatory cell infiltration of the graft wall was also revealed near the suture lines. Strands of myofibroblastic cells occasionally spread deep into the conduit wall from the adventitial side.

The luminal surface of Contegra was covered by an uneven neointimal layer without the involvement of the cusps. The maximum thickness of the neointima was found in the area of the distal anastomosis (~1070–1740 µm), and elsewhere it was in the range of ~130–760 µm. In the conduit, explanted after 1.6 months of follow-up (from the pig that died from non-conduit-related causes), a spread of the neointimal layer was noted in the area of both anastomoses, in the central part of the conduit devoid of fibrous tissue. Fibroblasts were present in the neointimal layer in all grafts, predominantly in the anastomotic area. A monolayer of endothelial cells partially covered the neointima at the distal and proximal anastomotic sites. The adjacent native PA preserved the endothelial layer. The Contegra cusps appeared preserved, with the correct fiber direction. In two cases, fibrin deposits and thrombi were revealed on both inflow and outflow surfaces of the valves. Cusp calcification was not observed.

The GA-BJV wall showed signs of edema with fragmented fibers. The wall was infiltrated with inflammatory cells (lymphocytes neutrophils, granulocytes, multinuclear macrophages, and a small number of eosinophils). Inflammation was most pronounced at the anastomotic sites and on the border of the neointima. Substantial calcification was revealed in the GA-BJV wall with the largest calcium deposits near the suture lines (Figure 4C). The area of calcification was related to the elastin fibers (Figure 4F,I). Neointimal tissue covered the inner surface of the GA-BJV unequally; it was absent in the central area and well defined at anastomotic sites (~770–1380 µm). Fibroblasts were observed in the neointima near the luminal surface and suture lines. The endothelium was absent from the inner surface of the GA-BJV grafts (Figure 4L). The leaflet structure was preserved; however, the fibers were fragmented. Mineralization foci were observed near the bases of the valve cusps. Foci of resolving inflammation with the formation of a fibrous capsule and neoangiogenesis were revealed in the adventitia. Native PA was infiltrated with inflammatory cells; it had a preserved endothelial layer, and calcifications were present at the anastomotic sites.

Three conduits from the animals with endocarditis were found to contain numerous bacterial or fungal colonies. In the conduit wall, degenerative changes were observed, characterized by a reduction in collagen and elastic fibers and productive inflammation with an abundance of neutrophils and macrophages. Granulation tissue with neovascularization, sclerosis, lysis, lymphocytic and histiocytic infiltration were revealed in the anastomotic zones. Thrombi with widespread inflammatory infiltration and colonies of bacteria and fungi were adjacent to the luminal surfaces of the grafts and leaflets, with both inflow and outflow surfaces. The valve matrix showed degenerative changes and lymphocytic cell infiltration. Necrotic foci were observed at the bases of the cusps.

### 3.3. Scanning Electron Microscopy

**Figure 7:**
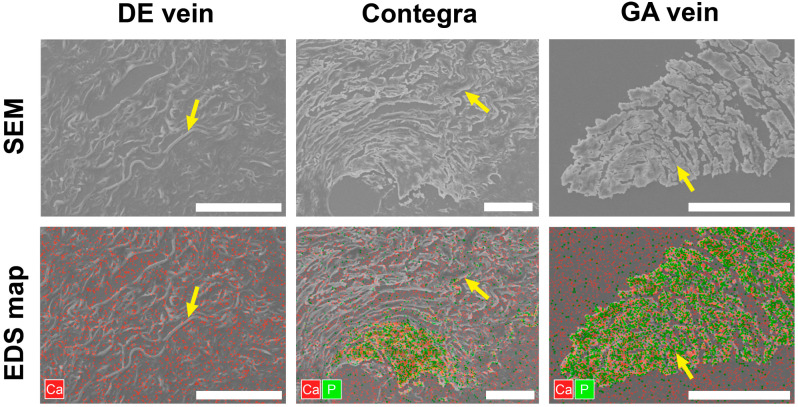
SEM images and EDS maps of dried sections. Yellow arrows indicate elastin fibers. Bars—50 μm.

The SEM imaging and elemental mapping of the observed fields revealed that the DE-treated samples were free from mineral deposits throughout the section. The detected calcium level matched the baseline level, whereas the signal corresponding to phosphorus levels was too weak to be accurately reflected on the map (Figure 7). Large crystal clusters with high calcium and phosphorus contents were found in the GA-treated samples. Although these conglomerates had hard mineral structures, their shapes resembled the direction of the elastin fibers in the replaced tissue (Figure 7). Similar picture were seen in the Contegra conduits. According to the EDS maps and reference scan images, the sites of mineralization were spread along the elastin fibers (Figure 7). Calcium to phosphorus ratio values for clusters in Contegra and GA-BJV were >1.5:1, which defines them as apatites.

## 4. Discussion

We assessed the structural changes in DE-BJV grafts and GA-cross-linking conduits implanted in the PA position in the pig model. In this study, the DE-BJV showed acceptable performance during the 6-month follow-up period. These conduits retained a smooth and clean inner surface without thrombosis, significant degeneration, or the calcification of the graft walls and leaflets. The DE-BJV leaflets were not deformed and their structures were preserved.

In our experimental series, DE-BJV conduits showed good graft integration. All DE-BJV grafts were characterized by the remodeling of the prosthesis tissue with the equal germination of the wall by myofibroblastic cells, the replacement of collagen fibers with soft non-deforming connective tissue, and active angiogenesis. Moreover, the migration of fibroblasts into the leaflet was noted in some grafts. In Contegra conduits, the germination of myofibroblasts extending from the adventitia into the depth of the wall was less pronounced. A dense fibrous capsule with remaining foci of resolving inflammation was detected in the adventitial layer of the GA-BJV. However, there were few myofibroblasts in the graft walls. We believe that these findings are due to DE toxicity, which was lower than GA toxicity [17,19,25]. The cytotoxic properties of GA have been previously described [11,12,19,26]. The formation of a fibrous capsule around the GA-BJV may play a role in isolating the graft from the surrounding tissues.

Cytotoxic properties of the treatment may also be reflected in the degree of endothelialization of the luminal surface of the graft. In our study, a well-developed continuous endothelial layer was found in all DE-BJVs (Figure 4J) but not in the GA-preserved conduits. Endothelial cells were differentiated via Russel–Movat pentacrome staining. In the Contegra conduits, the endothelial single layer was fragmentarily present in the proximal and distal anastomotic sites, which is consistent with previous findings [27]. Endothelial cells were completely absent from the luminal surface of the GA-BJV conduits (Figure 4L). GA released from the prosthesis tissues may inhibit the growth and metabolism of endothelial cells [13,14,28,29]. Simultaneously, DE had no cytotoxic effects on endothelial cell cultures, corroborating the results obtained [25]. Similar results have been demonstrated in studies on biomaterials cross-linked with polyepoxy compounds [30,31]. A normally developed and well-functioning endothelium prevents neointimal hyperplasia by suppressing its initial triggers, inhibiting the proliferation and migration of intimal smooth muscle cells, and decreasing the inflammatory response and clot formation [32].

The tendency toward neointimal hyperplasia was reduced in the DE-BJV conduits, compared to that in the GA-BJV, in our cohort. Most DE-BJVs had an even neointimal layer, with slight thickening at the suture lines. Neointimal hyperplasia at the distal anastomosis was noted in only two DE-BJV grafts. In both conduits, we observed the accumulation of foreign-body giant cells in the adventitia near the distal anastomosis and in the sub-neointimal region. In our opinion, the reaction to a foreign body in both conduits indirectly provoked neointimal proliferation due to the deformation of the conduit wall in the areas of giant-cell granulomas, and dense fibrous tissue formed along their periphery. We believe that the signs of reaction to a foreign body observed in both cases were a consequence of individual immune responses in these animals. Moreover, we did not observe any signs of reaction to a foreign body in other explanted DE-BJV conduits, which supports the impact of the immune response characteristics of each animal.

The explanted Contegra was characterized by a less even neointimal layer, deforming the wall of the conduit, and hyperplasia at the distal anastomotic site. Manifestations of neointimal hyperplasia were detected as early as 1.6 months after Contegra implantation. In GA-BJV conduits, the neointima did not completely cover the inner surface, hyperplasia was noted at the anastomotic sites, and the neointimal layer was completely absent in the middle part of the conduit. We attributed the neointimal hyperplasia in these groups to another manifestation of the cytotoxic effects of GA. On the one hand, a thicker intimal layer seems to be required to isolate the biomaterial containing residual GA [33]. On the other hand, GA cytotoxicity significantly inhibits cell migration and viability [11,26], preventing the formation of a continuous neointimal layer in the GA-BJV during follow-up.

Furthermore, the mechanical and hydrodynamic mechanisms at the anastomotic sites of the conduits may provoke neointimal hyperplasia [34,35]. This assumption was supported by the uneven thickness of the neointimal ridge along the distal anastomosis. The hyperplastic neointima was most pronounced along the lesser curvature of the distal part of the conduit. Peivandi et al. reported on the relationship between hyperplasia of the neointimal layer and the degree of elastin degradation, without any correlation with the duration of Contegra implantation in the PA position [36]. The authors concluded that the increasing degeneration of the elastin fiber network led to the progressive stiffness and rigidity of the graft, which contributed to neointimal hyperplasia. We did not obtain similar results in our experiments. Explanted BJV conduits, with and without neointimal hyperplasia, generally demonstrated retained elastin fibers.

Our previous experimental studies using a subcutaneous rat model showed that the degree of calcification of the BJV wall can be reduced by substituting GA with DE [15]. According to previous studies, GA-treated tissues are prone to early calcification [6,12,15]. The GA-BJV has demonstrated extensive calcium deposition in the wall as early as 3 months after implantation in sheep [37,38]. In clinical practice, over 60% of the explanted Contegra conduits had a pronounced calcification of its structures, including diffuse lesions [36]. Simultaneously, epoxy compounds significantly reduce the degree of calcium accumulation in biomaterials [10,15,16,30]. This experimental series confirmed a significantly lower tendency of mineralization in DE-BJV conduits in either Contegra or GA-BJV grafts. In our series, the DE-BJV wall was free of calcified lesions, and only a few prostheses had small calcium deposits at the suture sites. At the same time, calcium deposition was observed in the PA wall along the suture lines. We concluded that the suture material triggered calcification in this area. The GA-treated conduits were characterized by significantly more pronounced mineralization. In our series, Contegra and GA-BJV grafts had both large mature calcium deposits and foci of calcification along the course of elastin fibers (Figure 4). Moreover, calcified elastin fibers were detected in the Contegra conduit explanted after 1.6 months of follow-up. Our data further confirm that the mineralization of a BJV treated with GA begins with elastin fibers [15]. This finding was further supported by the SEM images and elemental analysis. The elemental maps gathered from the surface of the dried samples showed that the sites of calcium accumulation resemble the structure of elastin fibers, which could be differentiated on the scan images. SEM analysis also revealed the presence of large amounts of phosphorus in these mineral clusters, suggesting that they are apatite crystals (Figure 7). In addition, in our series, the mineralization of the GA-BJV was more extensive than that of Contegra, which may reflect the effect of a commercial storage solution [18,39].

Our previous study applying a subcutaneous rat model demonstrated that the DE treatment of a BJV inhibits the calcification of collagen, but does not affect elastin mineralization [15]. In this study, we found no evidence of calcium accumulation in either collagen or elastin in the DE-BJV wall after a 6-month follow-up period. This finding suggests that DE nevertheless contributes to slower elastin calcification compared to GA. The discrepancy between the results of the aforementioned studies is due to the different animal models.

Thrombosis of the DE-BJV conduit was not observed. Contegra conduits were characterized by the presence of thrombotic deposits, but this did not compromise the performance of the prosthesis. In an experimental series of GA-BJV conduits, thrombosis was detected in 18% of experimental animals after 8 months [40]. Our data support the antithrombogenicity of the DE-BJV. Epoxy compounds impart greater hydrophilicity to biomaterials, which leads to a decrease in tissue thrombogenicity [10,30,41]. In addition, DE-BJV conduits achieved good endothelialization in our series [32]. However, we preventively prescribed anticoagulants and antiplatelet therapy to all animals because of the tendency of pigs to have hypercoagulability [42,43], which also reduced the risk of thrombosis in our series.

We noted that the Contegra group was characterized by hyalinosis, and subintimal chondroid metaplasia with signs of calcification both in the graft wall and PA in the anastomotic area (Figure 4B and Figure 6). These transformations were confirmed via Von Kossa staining and IHC staining for S100 protein [44,45,46]. Previously, Peivandi et al. described foci of heterotopic ossification in Contegra conduits 9 years after implantation [36]. Chondroids and bone metaplasia have also been detected in other biomaterials treated in various ways [33,47], such as aortic allografts [48], stenotic native heart valves [49,50], and biodegradable polymeric vascular grafts [51,52,53]. Myofibroblasts, which differentiate into chondrocytes, may play a key role in the phenomenon of chondroid metaplasia and subsequent heterotopic ossification [33,50]. However, the reasons for this differentiation remain unclear. Inducing factors include local hypoxemia, due to the increased stability of hypoxia-inducible factor-1α, the expression of bone morphogenetic proteins, vascular endothelial growth factor and neuropilin-1, and inflammation with the release of cytokines that promote cartilage proliferation, as well as mechanical cues [33,49,54,55]. Subsequently, cartilage tissue can be replaced with heterotopic bone [49,50,55]. The pathogenesis of hyaline production entails the infiltration of plasma proteins like apolipoprotein E, Ig G, α2-macroglobulin and fibrinogen [56], which accumulate within the vascular wall, and induce the formation of fibrinoid conglomerates with subsequent hyalinization. These processes represent the consequence of the increased permeability of the vascular wall. Aseptic inflammation, attendant to the implantation of a sterile xenogenic graft, further increases the probability of the aforementioned scenario. Herein, hyalinosis and chondroid metaplasia were found only in animals with implanted Contegra grafts. Simultaneously, pathological foci were localized subintimally in the conduit, and at the anastomotic site in the PA. No similar changes were observed in other BJV conduits. Considering the present and previous findings, we propose that the possible inducing factors were a combination of material processing components. However, further studies are required to elucidate these mechanisms and their role.

Previous studies have reported a high incidence of endocarditis in BJV conduits treated with GA, which limits their use [2,8,9]. In our experiment, endocarditis developed in three animals during the observation period (DE-BJV—1; Contegra—1; GA-BJV—1). We cannot extrapolate the data on the incidence of endocarditis in our animal series to that associated with the use of DE-BJVs in humans because the animal model has hygiene and wound care limitations. It is difficult to adequately treat the postoperative suture under sterile conditions for an awake animal and to prevent damage to it in a postoperative aviary. Endocarditis occurred in most experimental series of conduit implantations in the pulmonary position in animal models [37,38,57]. However, good endothelialization of the conduit may reduce the risk of subsequent endocarditis in humans.

This was an experimental animal study, and its findings cannot be fully extrapolated to humans. However, the similarity of cardiac anatomy and hemodynamics allows us to build a concept of the functioning of the conduit from the DE-BJV in humans. It should be noted that we cannot fully project our results to groups of patients with high pulmonary hypertension or anatomical anomalies of the pulmonary arteries, because we implanted the conduit in healthy animals with well-developed pulmonary vessels. Another limitation of our study was the 6-month follow-up, but we identified structural differences between the conduits by the end of this follow-up period.

## 5. Conclusions

The DE-BJV conduits showed encouraging results in a porcine model for up to 6 months and are an acceptable alternative to GA-treated grafts. DE-BJV conduits demonstrated good integration without the development of significant degenerative changes. Good endothelialization of the inner surface, a low tendency of thrombosis, and calcium accumulation in the wall and leaflets are the advantages of these grafts. Meanwhile, Contegra and the GA-BJV had a tendency toward mineralization and had foci of calcification mainly along the course of elastin fibers. In GA-treated conduits, the endothelial layer was either fragmented or absent. In addition, foci of hyalinosis and chondroid metaplasia were found in the Contegra wall and adjacent PA; further studies on this phenomenon will help elucidate these processes.

The present findings suggest that the treatment of the BJV with DE reduces the risk of dysfunction and increases the durability of the conduit for RVOT reconstruction. Thus, DE-BJV conduits may improve the outcomes of surgeries for complex CHD in children. However, further experimental and clinical studies are required to comprehensively evaluate the performance of the DE-BJV.

## Figures and Tables

**Table 1:** Baseline and intraoperative characteristics.

Parameter	Me (IQR)
Animal gender, ♂/♀, n (%)	9/11 (45.0%/55.0%)
Weight at surgery, kg	46 (37.1–58.5)
Age at surgery, year	1 (0.83–1)
BJV diameter, n (%):	
12 mm	1 (5.0%)
14 mm	9 (45.0%)
16 mm	7 (35.0%)
18 mm	3 (15.0%)
Type of cannulation:	
central, n (%)	12 (60.0%)
peripheral, n (%)	8 (40.0%)
CPB time, min	60 (45–65)
Mechanical ventilation, h	5.5 (4.0–5.5)

BJV, bovine jugular vein; CPB, cardio-pulmonary bypass; Me, median; IQR, interquartile range.

## Data Availability

The data presented in this study are available within the article.
